# Advances in Adenomyosis Treatment: High-Intensity Focused Ultrasound, Percutaneous Microwave Therapy, and Radiofrequency Ablation

**DOI:** 10.3390/jcm13195828

**Published:** 2024-09-29

**Authors:** Adamantios Athanasiou, Arrigo Fruscalzo, Ioannis Dedes, Michael D. Mueller, Ambrogio P. Londero, Carolin Marti, Benedetta Guani, Anis Feki

**Affiliations:** 1 Department of Gynecologic Oncology, Agios Savvas General Anti-Cancer Hospital of Athens, 11522 Athens, Greece; diamathan16@gmail.com; 2 Department of Obstetrics and Gynecology, University Hospital of Fribourg, 1708 Fribourg, Switzerland; carolin.marti@h-fr.ch (C.M.); benedetta.guani@h-fr.ch (B.G.); anis.feki@h-fr.ch (A.F.); 3 Department of Obstetrics and Gynecology, University Hospital of Bern, 3010 Bern, Switzerland; ioannis.dedes@insel.ch (I.D.); michel.mueller@insel.ch (M.D.M.); 4 Department of Neuroscience, Rehabilitation, Ophthalmology, Genetics, Maternal and Infant Health, University of Genoa, 16132 Genoa, Italy; ambrogiopietro.londero@unige.it; 5 Obstetrics and Gynecology Unit, IRCCS Istituto Giannina Gaslini, 16147 Genova, Italy

**Keywords:** adenomyosis, therapy, high-intensity focused ultrasound (HIFU), percutaneous microwave ablation (PMWA), radiofrequency ablation (RFA)

## Abstract

**Background/Objectives**: Adenomyosis is a debilitating gynecologic condition that affects both multiparous older women and nulliparous younger women, inducing a variety of symptoms such as dysmenorrhea, menorrhagia, and infertility. Thermal ablation techniques are new procedures that have been proposed for the treatment of adenomyosis. They include high-intensity focused ultrasound (HIFU), percutaneous microwave ablation (PMWA), and radiofrequency ablation (RFA). Because thermal ablation techniques are minimally invasive or noninvasive, fertility is not impaired while symptoms improve. In addition, hospital stays and financial costs are generally reduced, increasing the interest in these alternative management options. **Methods**: In this narrative review, we conducted a thorough literature search of PubMed/Medline from the database inception to September 2022. In our search, we focused on noninvasive treatment methods such as HIFU ablation, RFA ablation, and PMWA as well as adenomyosis-specific terms and noninvasive techniques (ultrasonography, ultrasound, or magnetic resonance imaging). The queries were a combination of MeSH terms and keywords. The search was limited to the English language. Abstracts were screened according to their content, and relevant articles were selected. **Results**: Overall, the results showed that the above-mentioned ablation techniques are effective and safe in providing adenomyosis treatment. Lesion size and uterus volume are reduced, leading to considerable symptom alleviation with all three methods. Positive results concerning safety and fertility preservation have been described as well. **Conclusions**: Nonetheless, more research is required in this field to compare the efficacy and safety of different ablation techniques with traditional therapies. Such research will help improve these procedures and their associated decision-making processes.

## 1. Introduction

Adenomyosis is a condition involving glands (from the Greek αδ*ϵ*ν*ϵ*ς = adeno) and muscles (from the Greek µυς = myosis) [1]. The current scientific definition, primarily based on that provided by Bird et al. [2], describes adenomyosis is a “benign invasion of the endometrium into the myometrium producing a diffusely enlarged uterus which microscopically exhibits ectopic, non-neoplastic, endometrial glands surrounded by hypertrophic-hyperplastic musculature”. It is now recognized that adenomyosis affects not only multiparous older women, as was historically believed based on histopathological diagnosis following hysterectomy, but also nulliparous younger women [3]. Although the exact etiopathogenesis of adenomyosis remains uncertain, several hypotheses exist [4,5]. One prominent theory suggests that intense uterine contractions during menstruation cause repeated microtrauma to the endometrial basal lamina, leading to the invagination of endometrial tissue into the myometrium [4,5]. This process, referred to as endometrial–myometrial interface disruption, may be driven by inflammation, local estrogen production, epigenetic changes, or somatic mutations. Other proposed mechanisms include metaplasia of embryonic Müllerian remnants, differentiation of embryonic stem/progenitor cells, and extension of peritoneal pelvic endometriosis into the myometrium [4,5] Additionally, increased stiffness of the internal cervical os may contribute to adenomyosis by obstructing menstrual flow and enhancing uterine contractions, thereby facilitating endometrial invasion into the myometrium [4,5].

Adenomyosis is a common gynecological condition affecting women, especially those of reproductive age. The prevalence varies according to the population type, ranging from approximately 20% to 35% in nonemergency outpatient settings [6,7]. In one cross-sectional study, adenomyosis was found in 86.1% of women who presented with gynecological symptoms in the emergency department, making it the most common pathology among these patients [8]. According to that study, adenomyosis was prevalent in all age groups but more common in women ≥41 years of age [8]. This prevalence highlights the significant impact of adenomyosis on women’s health as well as the importance of early diagnosis and treatment.

The introduction of imaging techniques such as transvaginal ultrasound and magnetic resonance imaging (MRI) has resulted in diagnostic advancements. Transvaginal ultrasound and MRI can diagnose adenomyosis accurately without invasive procedures, which is critical for guiding appropriate clinical management [9,10]. If adenomyotic lesions in the myometrium are examined according to their pattern, we can distinguish between focal and diffuse adenomyosis [11]. In some adolescents and young adult patients, the disease presents as a cystic adenomyoma [12]. Even though, several classifications of adenomyosis into subtypes according to the findings of histopathology and imaging have been proposed. Still, there is no consensus on which one should be universally used [11].

A transvaginal ultrasound scan is a relatively easy-to-perform first-line method for a reliable diagnosis of adenomyosis, while magnetic resonance should be proposed as a second-line method. According to a recent meta-analysis, ultrasound scans reached a 78% value for both specificity and sensitivity [10]. In 2015, an expert panel, the MUSA (Morphological Uterus Sonographic Assessment) group, developed, for the first time, a consensus on terms, definitions, and measurements to describe and report the sonographic features of adenomyosis. These criteria were further refined in 2019 and subdivided into two categories: direct signs (myometrial cysts, hyperechogenic islands, and echogenic subendometrial lines and buds) and indirect signs of adenomyosis (globular uterus, asymmetrical myometrial thickening, fan-shaped shadowing, translesional vascularity, irregular junctional zone, and interrupted junctional zone) [10].

Regarding the clinical presentation, adenomyosis exhibits symptoms of varying intensity, infertility, and connection with endometriosis [13]. Common symptoms are intense pelvic pain, dysmenorrhea, menorrhagia, and infertility; however, asymptomatic cases also exist. An investigation of the recent literature reveals that no definitive conclusions have been reached regarding either the pathogenesis or the diagnostic criteria, leading to a great need for further research. Evidence concerning the efficacy of the different treatment options is also limited despite the fact that research in this field is steadily progressing [14,15].

The standard treatment for adenomyosis is currently considered to be surgical. So far, hysterectomy can be proposed as a curative therapeutic option but it is invasive and, unfortunately, unsuitable for females who want to retain their reproductive capability. Consequently, physicians resort to conservative surgical resection, preserving healthy remaining tissue. Nonetheless, surgical techniques could be very challenging and can expose the women to potentially severe complications in case of future pregnancy, like uterine rupture [15]. Alternative medical treatments can be proposed, including oral contraceptive pills, levonorgestrel-releasing intrauterine system (LNG-IUS), gonadotropin-releasing hormone analogs (GnRH-a), and further compounds still less investigated [14]. However, their efficacy seems to be still limited, particularly in some therapy-refractory cases. In this context, nonsurgical ablative techniques like thermal ablation procedures can potentially play a role as a game-changer in the conservative treatment of adenomyosis.

The objective of this review is to present an overview of the current literature on thermal ablation procedures for the noninvasive treatment of adenomyosis. Techniques reviewed include high-intensity focused ultrasound (HIFU), percutaneous microwave ablation (PMWA), and radiofrequency ablation (RFA). The feasibility, treatment success, safety, benefits, and drawbacks of each therapy are evaluated.

## 2. Methods

The literature research was focused on noninvasive ablative methods for the treatment of adenomyosis, specifically concerning HIFU, RFA, and PMWA procedures. Women seeking a nonsurgical uterine-sparing treatment were the target population. Outcomes data searched for included feasibility, efficacy, safety issues, and fertility. Data were compared, when dates were available, with current therapies for adenomyosis, both surgical and medical ones.

For the purpose of this narrative review, a literature search of PubMed and Medline was performed from the database inception to September 2022 using the following keywords: “adenomyosis” [MeSH Terms] AND “high-intensity focused ultrasound ablation” [MeSH Terms], “adenomyosis” [MeSH Terms] AND “radiofrequency ablation” [MeSH Terms], “adenomyosis” [MeSH Terms] AND “microwaves” [MeSH Terms], “adenomyosis” AND “therapy”, “adenomyosis AND histology”, “adenomyosis AND imaging”, “adenomyosis AND ultrasonography OR ultrasound”, “adenomyosis AND magnetic resonance imaging”. The search was limited to the English language. Two independent authors (DA and AFr) screened abstracts according to their content, and relevant articles were selected.

This review was performed following the SANRA (scale for the quality assessment of narrative review articles) recommendations, developed to improve the quality of narrative reviews [16]. The following six items were retained in order to select the sources of the literature with a higher standard of quality: an explanation of (1) the importance and (2) the aims of the review, (3) the literature search and (4) referencing and presentation of (5) the evidence level, and (6) relevant endpoint data. A total of 134 original research papers and review articles were identified. We selected 62 relevant prospective cohort studies, randomized controlled trials (RCTs), and review articles addressing the topic of thermal ablation procedures for the noninvasive treatment of adenomyosis.

## 3. Results—General Principles of Thermal Ablation Methods

Beyond the option of hysterectomy and fertility-sparing surgical treatments for adenomyosis, image-guided, thermal ablation, and minimally invasive procedures are currently showing promise in managing symptomatic adenomyosis and are considered safe and effective. Three focused tissue thermal ablation technologies are used: HIFU, PMWA, and RFA.

**Figure 1:**
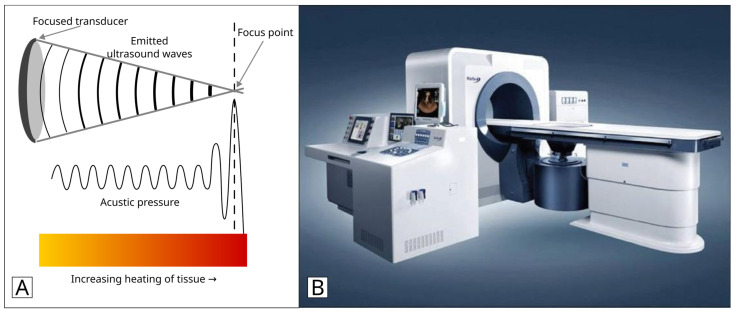
(**A**): Schematic representation of ultrasound-guided high-intensity focused ultrasound (HIFU) mechanism of action; (**B**): JC model of HIFU tumor therapeutic system [19].

HIFU is a local, noninvasive thermal ablation technique. A concave extracorporeal transducer generates the ultrasound beams. It is guided by MRI or ultrasonography, and its action affects the target area thermally, cavitationally, and mechanically (Figure 1). While treatment is active, the ultrasound beams enter the tissue, especially via the acoustic route, and are then guided to the body’s target region. At temperatures of >65 °C, the target tissue undergoes coagulative necrosis [17]. Despite the expense of MRI, there are advantages in real-time temperature mapping and high anatomical resolution. Additionally, ultrasound guidance provides real-time anatomic imaging for monitoring the treatment response and is less costly [18].

**Figure 2:**
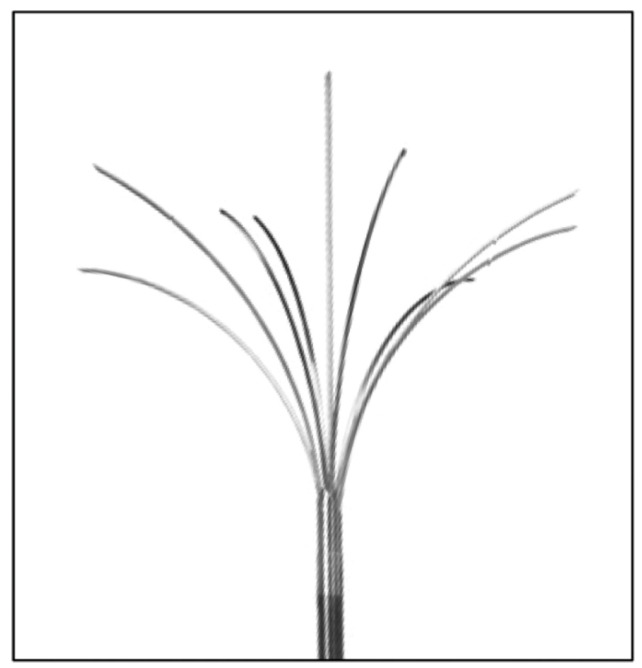
A radiofrequency needle electrode with extendible prongs, used for uterine nodular adenomyosis ablation [21].

The second therapy is ultrasound-guided RFA. Under ultrasound guidance, a needle is inserted directly into the lesion, and the heat produced affects the target lesion (Figure 2). A high-frequency alternating electrical current generates heat by ionic friction, achieving tissue necrosis [20].

**Figure 3:**
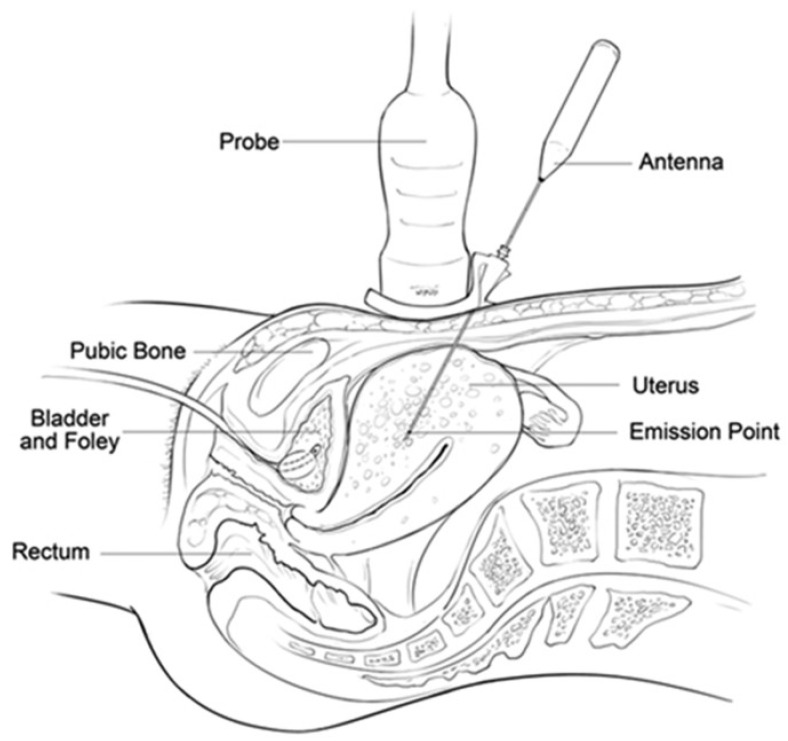
Ultrasound-guided percutaneous microwave ablation for adenomyosis [23].

The third therapy is PMWA. PMWA is a percutaneous, in situ therapy that works through ultrasound guidance (Figure 3). Its purpose is to induce coagulation necrosis of the lesion through heat [22]. PMWA uses electromagnetic energy to rapidly rotate polar water molecules next to each other to generate heat. Its mechanics depend on the percutaneous insertion of an electrode directly into the lesion under ultrasound guidance [20].

A summary of studies on three ablation techniques for adenomyosis (HIFU, PMWA and RFA) is provided below.

### 3.1. HIFU (Table 1)

HIFU is probably the most consolidated noninvasive thermal ablation technique. A summary of studies concerning feasibility, efficacy, safety and drawbacks on this ablation technique for adenomyosis is provided below (Table 1).

**Table 1:** Summary of studies on HIFU ablation technique for adenomyosis.

High-Intensity Focused Ultrasound				
Article Title	First Author, Year	Study Type and Location	Number of Patients	Main Results
Clinical effectiveness and potential long-term benefits of high-intensity focused ultrasound therapy for patients with adenomyosis	Li, 2020 [24]	Retrospective study China	67 patients for 2 years	HIFU provided considerable relief for patients with severe preoperative dysmenorrhea. Patients with a high pre-HIFU NRS score had good results. The effectiveness of HIFU for reducing uterine lesion volume and hypermenorrhea was marginal.
Clinical predictors of long-term success in ultrasound-guided high-intensity focused ultrasound ablation treatment for adenomyosis	Liu, 2016 [25]	Retrospective study China	230 patients	The study focused on the long-term (>3-year) value of HIFU ablation treatment. The long-term success rate was satisfactory. Patients with a higher NPV ratio and older age had higher success rates. The recurrence rate was higher in patients with a higher BMI and lower acoustic power.
Factors influencing the ablative efficiency of high-intensity focused ultrasound (HIFU) treatment for adenomyosis	Gong, 2016 [26]	Retrospective study China	245 patients	Factors that rendered HIFU therapy for adenomyosis less effective are as follows: greater abdominal thickness, greater distance from the ventral side of the lesion to the skin, degree of arterial enhancement, and amount of high T2 signal in a lesion.
Changes in anti-Müllerian hormone levels as a biomarker for ovarian reserve after ultrasound-guided high-intensity focused ultrasound treatment of adenomyosis and uterine fibroid	Lee, 2017 [27]	Retrospective study China	79 patients	HIFU is a safe and effective treatment option for reducing symptoms of adenomyosis, including dysmenorrhea and menorrhagia. It is a better therapeutic option than LE for patients with adenomyosis and infertility, resulting in higher conception rates and live birth rates.
High-intensity focused ultrasound (HIFU) combined with gonadotropin-releasing hormone analogs (GnRHa) and levonorgestrel-releasing intrauterine system (LNG-IUS) for adenomyosis	Haiyan, 2019 [28]	Case series with long-term 5-year follow-up China	142 patients conservatively treated with HIFU in combination with adjuvant GnRH-a and LNG-IUS	Both the uterine and lesion volumes significantly decreased after treatment. The average reduction rate was 45% at 12 months followed by an increase of 35%. AD lesion volumes, dysmenorrhea, and menstrual flow showed similar decreases. Hemoglobin levels increased. Recurrence rates remained low.
High-intensity focused ultrasound in the management of adenomyosis: long-term results from a single center	Li, 2021 [29]	Retrospective study China	1982 patients: 485 → only HIFU 289 → HIFU followed by GnRH-a 594 → HIFU combined with GnRH-a and Mirena	After HIFU ablation, the dysmenorrhea severity pain score and the menorrhagia severity score were considerably decreased at each follow-up point. As the follow-up time increased, the effective rate of HIFU treatment in improving dysmenorrhea and menorrhagia decreased. At the 6-month and 3-year follow-ups, the efficacy of HIFU + Mirena and HIFU + GnRH-a + Mirena was significantly higher than that of HIFU alone and HIFU + GnRH-a.
Effect of pretreatment with gonadotropin-releasing hormone analog GnRH-α on high-intensity focused ultrasound ablation for diffuse adenomyosis: a preliminary study	Xiao-Ying, 2018 [30]	Retrospective cohort study China	61 patients in two groups: 23 patients with larger uteri received pretreatment with GnRH-a and were then subjected to HIFU; 38 patients received HIFU only	Although the lesion volume in the HIFU + GnRH group was larger than that in the HIFU-only group, the study revealed higher NPV, NPVR%, treatment intensity, and total energy with shorter treatment and sonication times in the HIFU + GnRH group than in the HIFU-only group. Noticeable differences were observed for NPV, NPVR%, average power, and total intensity energy between the two groups, but not for other parameters. HIFU + GnRH was more effective than HIFU alone for the ablation of diffuse AD. The GnRH pretreatment was safe.
Efficacy of high-intensity focused ultrasound combined with GnRH-a for adenomyosis	Pang, 2021 [31]	Systematic review and meta-analysis China	Data from 766 patients with AD were studied	HIFU + GnRH-a for the treatment of adenomyosis had greater efficacy in decreasing the volumes of the uterine and adenomyotic lesions and alleviating symptoms. More studies are needed to verify the long-term efficacy of the treatment.
Feasibility of MRI-guided high intensity focused ultrasound treatment for adenomyosis	Fan, 2011 [17]	Clinical trial China	10 patients	The trial results acknowledged that HIFU treatment is a feasible and safe ablation method that also relieves symptoms.

AD: Adenomyosis; HIFU: high-intensity focused ultrasound; GnRH-a: gonadotropin-releasing hormone analog; LNG-IUS: levonorgestrel-releasing intrauterine system; NPV: non-perfusion volume; NPVR: NPV ratio.

#### 3.1.1. HIFU: Feasibility

With respect to the feasibility of HIFU, problems have been encountered in patients with increased abdominal wall thickness, lesions located in the posterior uterine wall, and lesions with an excessive blood supply. These issues arise because the thermal energy may have difficulty reaching the lesion due to the increased distance or may be dissipated by the increased blood supply. The feasibility and efficacy of HIFU depend on several factors, including the uterine position, the volume and vascularization of the adenomyotic lesions, the distance to the lesion, and the abdominal wall thickness, which should ideally be ≤1 cm [19].

#### 3.1.2. HIFU: Efficacy

Several original studies have focused on the efficacy of HIFU. They have all confirmed the effectiveness of this conservative approach regarding various outcomes, including both clinical outcomes (hypermenorrhea, dysmenorrhea, and fertility) and anatomic landmarks (uterine dimensions or lesion volume). Two similar retrospective studies performed in China by Li et al. [24] and Liu et al. [25] focused on the long-term efficacy of HIFU for the treatment of adenomyosis. Both studies showed that dysmenorrhea was significantly decreased post-treatment, whereas hypermenorrhea was not [24]. Additionally, Li et al. [24] examined the uterine lesion volume, for which HIFU demonstrated only borderline effectiveness. Liu et al. [25] verified a link between the success of HIFU and the non-perfused volume (NPV) ratio according to the following four factors: adenomyosis type, adenomyotic lesion volume, mean acoustic intensity, and NPV. Moreover, an age of >40 years was found to contribute to the success of HIFU treatment. Both studies concluded that HIFU ablation is safe, effective, and clinically valuable [24,25]. A similar approach was used by Gong et al. [26] in their retrospective study, which was performed to investigate factors associated with the ablative efficiency of HIFU. The authors found that wall thickness and lesion location were related to the energy efficiency factor (EEF), NPV ratio, and grayscale changes during treatment. Patients with thick abdominal walls or lesions in the posterior wall exhibited a significantly higher EEF and lower NPV ratio and image pattern changes. Moreover, patients with more extensive lesions showed a significantly higher NPV ratio combined with a considerably decreased EEF. An association was also found between hyper-enhancing lesions and an excessive blood supply, making HIFU treatment more difficult and resulting in a lower NPV ratio and a significantly higher EEF [26]. Another study showed that HIFU for the treatment of adenomyosis significantly reduced the uterine volume compared with pretreatment [27]. Results were even present at 3, 6, and 12 months of follow-up after treatment. Moreover, at these same post-treatment time points, the symptom severity score decreased while the Uterine Fibroid Symptom Health-Related Quality of Life Questionnaire score increased [27]. An investigation performed by Du et al. [32] focused on the association between the number of hyperintense foci on T2-weighted MRI and the efficacy of HIFU for adenomyosis treatment. The patients in this prospective cohort study were divided into two groups based on the number of hyperintense foci present before treatment (<5 or >5). Post-treatment follow-ups were scheduled at 6, 12, 24, and 36 months. The results showed that the treatment time, dose, and EEF were significantly higher and that the ablation rate was lower in the group with >5 hyperintense foci [32].

In their systematic review and meta-analysis, Marques et al. [33] investigated the efficacy of HIFU in decreasing adenomyotic lesion size, bleeding, and pain and increasing quality of life. They found a considerable decrease in the volume of both adenomyotic lesions and the uterus, and this effect lasted for 12 months post-HIFU. The rates of dysmenorrhea and menorrhagia from the pooled results were significantly reduced for up to 24 months post-treatment.

The most recent study was performed by Gong et al. in 2022 [34]. The authors assessed the improvement in the median menstrual pain score and the median menstrual blood volume at 18 months post-HIFU. HIFU alleviated both symptoms in all subtypes of adenomyosis. With respect to dysmenorrhea, patients with internal adenomyosis had the least intense pain, while patients with asymmetric external adenomyosis had the greatest pain at 18 months; however, no statistically significant difference in menstrual pain relief was observed among the subtypes. Regarding menorrhagia, HIFU improved the menstrual blood volume in all subtypes, although significantly less pain relief was achieved for the asymmetric external subtype than the others [34].

Finally, Huang et al. [35] compared the treatment impact of HIFU and laparoscopic excision among patients with adenomyosis. The author retrospectively evaluated the clinical outcome of 93 patients with adenomyosis and infertility, 50 of them treated with HIFU and 43 with laparoscopic excision. Both treatments considerably alleviated dysmenorrhea and menorrhagia. Furthermore, although neither patient group demonstrated critical post-treatment complications, the HIFU group had a shorter hospitalization duration. Concerning fertility, the HIFU group achieved considerably higher natural conception and pregnancy rates. In a similar context, patients with diffuse adenomyosis treated by HIFU had significantly lower post-treatment pregnancy rates than those with focal adenomyosis [35]. Equally important, no significant change occurred in the anti-Müllerian hormone (AMH) level, which reflects the ovarian reserve between pretreatment and 6 months post-treatment [27].

#### 3.1.3. HIFU: Combination with Other Treatments to Increase Efficacy

There is a new field of research concerning the positive effect of combined therapies for improving HIFU efficacy. Enhancing the efficacy of the treatment using the same or a lower degree of energy may reduce the adverse effects and risks of HIFU.

Yao et al. [36] investigated the synergistic positive effects of SonoVue (a microbubble-based ultrasound contrast agent) and oxytocin on the efficacy of HIFU. The researchers concluded that combining oxytocin and SonoVue reduced the EEF and limited the ablation time compared with using each agent alone. Moreover, the efficiency of HIFU increased due to improved cavitation and thermal mechanisms, as indicated by an increase in the post-treatment NPV [36].

A crucial development in HIFU treatment is its combination with other methods such as a levonorgestrel-releasing intrauterine system (LNG-IUS) or gonadotropin-releasing hormone agonist (GnRH-a). Four studies examined the effects of combined HIFU treatment and concluded that it was more effective than HIFU alone. Guo et al. [37] discovered that combining HIFU with GnRH-a therapy reduced the serum CA-125 level, the size of adenomyotic lesions in the uterus, and menstrual blood flow. Moreover, dysmenorrhea and clinical outcomes were improved in the short-term follow-up. However, the authors did not examine long-term effects. Haiyan et al. [28] expanded upon the above results by studying the long-term effects of HIFU in combination with an LNG-IUS and GnRH-a therapy. The results revealed that both the lesion and uterine volumes considerably decreased, exhibiting a 45% reduction rate at 12 months post-treatment. Dysmenorrhea and menstrual flow symptoms also decreased. Moreover, the long-term symptom recurrence rate also remained low during the 5-year post-treatment period. Similarly, Li et al. [29] retrospectively investigated the long-term clinical results of adenomyosis treated by HIFU (H) alone; HIFU and Mirena, an LNG-IUS (H+M); HIFU and GnRH-a (H+G); and HIFU with GnRH-a and Mirena (H+M+G) during a follow-up period of 5 years. Initially, the H treatment decreased dysmenorrhea and menorrhagia symptoms by >85% at 3 months post-treatment, while at 5 years, this rate of decrease was 65%. The symptom relief rate continued to decrease as follow-up continued. H+M and H+M+G treatments provided longer-term relief of symptoms, whereas H+G provided only short-term improvement [29]. A study conducted in China by Xiao-Ying et al. [30] in 2018 produced similar results, although the GnRH-a therapy was used before HIFU for diffuse adenomyosis in patients with a large uterus. Despite the higher lesion volume in the H+G group than in the H group, the results showed higher NPVs and NPV ratios and shorter treatment and sonication times in the H+G group. Moreover, the symptom relief rate improved more in the H+G group at 6 and 12 months post-treatment than in the H group. In a recent systematic review and meta-analysis, Pang et al. [31] evaluated the efficacy of HIFU with GnRH-a. Regarding the primary outcomes, the study showed smaller uterus and adenomyotic lesion volumes with the combined treatment. Additionally, all secondary outcomes (dysmenorrhea, menorrhagia, serum CA-125 level, and recurrence rate) showed greater improvement with the combination therapy than with HIFU alone.

#### 3.1.4. HIFU: Safety and Drawbacks

Evidence confirms that infrequent and mostly minor complications are encountered with HIFU. The reported incidence of complications after treatment is relatively low at 2.8%. The most commonly appearing symptoms are discomfort and pain in the treated region, lower abdominal pain, superficial skin burns, and vaginal discharge, although these can be self-treated [19,25]. Less frequent adverse effects include deeper skin burns, leg pain, and bowel perforation. Patients with surgical scars in the abdomen have a higher incidence of skin burns due to denervated tissue with consequent sensory loss in the skin. Bowel injury can be entirely avoided if patients follow the preparation protocol.

Although there is no conclusive evidence regarding the relationship between fertility and HIFU, precise lesion ablation prevents damage to the myometrium and endometrium and, therefore, prevents scar tissue formation. Compared with surgical intervention, HIFU produces better outcomes with respect to both post-treatment conception and the risk of uterine rupture [19]. Another study evaluating fertility conservation after HIFU showed no significant difference in the AMH level before HIFU ablation and 6 months post-treatment, indicating that the ovarian reserve remained unaffected [27].

According to more recent bibliography, there are possible complications such as thermal injuries that lead to skin necrosis in the uterus, the peritoneum, the anterior abdominal wall muscles, and the subcutaneous tissue. More serious complications, including internal organ damage and skin burns due to false targeting, exaggeration of power strength, mechanical causes or operators with insufficient skills and training have been described [38].

### 3.2. RFA

RFA is another thermal ablation technique for treating dysmenorrhea, menorrhagia, and related problems such as subacute and chronic anemia. Relatively few studies have focused on this noninvasive procedure. A recent review examined 7 studies involving 396 patients who underwent RFA treatment for symptomatic adenomyosis [39]. One was a high-quality prospective randomized controlled clinical trial, and the others were retrospective studies. A summary of studies on radiofrequency ablation technique for adenomyosis is provided below (Table 2).

#### 3.2.1. RFA: Feasibility

There is a wealth of encouraging data on RFA feasibility and efficacy. Scarperi et al. [21] conducted a feasibility study in which 15 women were treated for symptomatic nodular adenomyosis using laparoscopic RFA. Additionally, a recent randomized study compared RFA and microwave ablation [20]. The electrode was percutaneously inserted into the lesion in this trial using transabdominal ultrasound guidance. The procedure was successful in all 65 patients included in the study. The average ablation time was 37.5 ± 6.2 min, which appears to be relatively short for this procedure [20].

#### 3.2.2. RFA: Efficacy

Various outcomes have been proposed for evaluating RFA efficacy. One major outcome is the clinical improvement in symptoms such as dysmenorrhea and hypermenorrhea. Another outcome is the anatomical change in the appearance and dimensions of lesions. In the above-cited study by Lin et al. [20], dysmenorrhea was evaluated 12 months after ablation. In the RFA group, 4 (7.6%) patients showed no improvement in dysmenorrhea, 26 (49.3%) reported complete relief, and 23 (43.1%) reported partial relief.

Similar results were reported in the study by Scarperi et al. [21], who noted progressive improvement of dysmenorrhea at four follow-ups throughout a 12-month period. Accordingly, considerable symptom improvement was evident at all four follow-ups, with pain reduction rates of up to 40.0%, 57.5%, 68.1%, and 71.3% at 3, 6, 9, and 12 months, respectively [21]. Hai et al. [41] performed a retrospective study assessing the midterm results of transvaginal RFA for treating symptomatic uterine adenomyosis. They found notable uterine volume reduction rates of 35.8%, 40.8%, and 41.2% at 1, 6, and 12 months of follow-up, respectively. Additionally, there were statistically significant reductions in dysmenorrhea and further symptoms. During the procedure, no patients developed severe complications involving injury to adjacent organs, sepsis, or peritonitis. However, 2 of 87 patients developed post-ablation intrauterine adhesions [41].

Hai et al. [40] conducted another study to evaluate the clinical outcomes of transvaginal RFA combined with an LNG-IUS for treating symptomatic uterine adenomyosis. The LNG-IUS is effective, long-lasting, and reversible, and it is used for patients with a uterine length of <9 cm [40]. Overall, the combined treatment in their study was highly effective. Initially, the authors reported a 55% reduction in uterine size after one year of treatment. Furthermore, the dysmenorrhea and menorrhagia scores significantly decreased after ablation and remained low throughout the 3-year follow-up. Finally, no patients experienced symptom relapse during the 3-year follow-up [40].

Another efficacy-related issue to consider is the recurrence rate. One study found symptom recurrence in approximately 20% of treated patients [42]. In a recent long-term follow-up study on uterine-sparing treatment of adenomyosis with RFA in 60 hysterectomy candidates, RFA avoided hysterectomy in 87% of the patients [44]. Furthermore, significant improvements in pain symptomatology, uterine bleeding, and bulk symptoms were observed, indicating that this treatment can be beneficial over time [44].

Taking into consideration that there is no official, common protocol treatment for adenomyosis, it is vital to know how efficacious each treatment is. A recent case study investigated several treatment methods of adenomyosis patients in the context of clinical efficacy and fertility results compared to standard therapy [43]. The 140 participating patients were randomly separated into 4 groups: A, B, C, and D. Group A patients underwent laparoscopic surgery while those of group B were treated with laparoscopic surgery in combination with GnRH-a. In group C, RFA was used and finally, group D received RFA accompanied by GnRH-a. Evaluating the results in terms of menstrual volume, dysmenorrea score, uterine volume, clinical efficiency, LH, E2, FSH levels, CA125 levels, recurrence, pregnancy status, and outcomes, the combination of RFA with GnRH-a as used in group D achieved overall efficiency of 100%. It was followed by 71.43%, 80% and 82.86% in groups A, B, and C respectively [43].

Concerning fertility, in addition to the above-cited study of Chu et al., few studies have reported in depth the fertility outcomes after RFA [44,45]. Of note, an extensive series of patients was retrospectively examined by Nam [45]. Among 39 pregnancies in 29 patients, there were 24 deliveries, 12 spontaneous miscarriages, and 3 pregnancy interruptions. Among the 59 patients intending to conceive, the clinical pregnancy rate was 50%, with a spontaneous conception rate of 42.7% [45].

#### 3.2.3. RFA: Safety and Drawbacks

Relatively little research to date has focused on the safety and drawbacks of RFA. However, the data that have been accumulated are promising. In the study by Lin et al. [20], the most frequent adverse events after RFA were abdominal pain, vaginal discharge, and low-grade fever. Notably, these symptoms were self-limiting and disappeared within the first 2 weeks [20]. Regarding fertility, RFA may provide advantages over surgery, eliminating abdominal incisions, uterine scars, or bleeding. However, a drawback of this ablation technique is the inability to accurately monitor the temperature setting once the probe has been transvaginally implanted. This issue may lead to thermal injury to the endometrium, resulting in intrauterine adhesions. This is a disadvantage in patients who desire fertility preservation [41].

According to the current bibliography, RFA offers a minimally invasive treatment option for organ preservation. Furthermore, the existing databases still do not report serious complications. The most frequent complications reported include vaginal discharge, abdominal pain, and transient fever. Overall, RFA is regarded as a safe thermal ablation technique. Safety issues can be reduced by using the RFA option because it is considered less invasive than HIFU or MWA. Consequently, unnecessary tissue removal can be avoided, which, in turn, can decrease complications, including uterine rupture phenomena, during pregnancy [39].

### 3.3. PMWA

PMWA is probably the less consolidated noninvasive thermal ablation technique. A summary of studies concerning feasibility, efficacy, safety and drawbacks on this ablation technique for adenomyosis is provided below (Table 3).

#### 3.3.1. PMWA: Feasibility and Efficacy

PMWA is the most recently developed thermal ablation technique. Notably, the amount of energy required per unit volume of uterine lesions during the procedure remains unclear. A better estimation of the effect obtained will allow for a more accurate prediction of the amount of energy that must be applied before treatment, thereby helping to standardize the procedure. Contrast-enhanced ultrasonography and dynamic contrast-enhanced MRI can be used to evaluate the effect of PMWA on adenomyosis [49]. These techniques were found to be advantageous in accurately assessing the ablation rate, and researchers thus consider them worthy of clinical promotion. In fact, the impact of PMWA on localized adenomyosis can be dynamically observed in real time by contrast-enhanced ultrasonography. In addition, the contrast medium helps evaluate the post-treatment effect and ablation rate. Moreover, no adverse effects related to contrast agents have been reported. Patients report only pain in the treated region, which is easily controlled with a single dose of an analgesic [49].

A Chinese study conducted in Beijing focused on the efficacy of PMWA for treating adenomyosis [23]. The study examined the changes in the serum levels of pituitary hormones, gonadal hormones, CA-125, estradiol, follicle-stimulating hormone (FSH), and prolactin before ablation and 6, 9, and 12 months after ablation. The results revealed no significant differences in the estradiol and FSH levels before and after treatment. However, the mean CA-125 and prolactin levels were significantly lower at follow-up. High prolactin levels have been shown to induce the development of adenomyosis [50]. Moreover, the researchers found a significant association between changes in the CA-125 level and uterine volume, specifically noting a decrease in the pretreatment CA-125 levels in patients whose uteri were >240 cm^3^ pre-ablation. Adenomyosis has been linked to an increase in the CA-125 level, which is a nonspecific disease marker. In one study, PMWA had satisfactory clinical results and a low rate of complications except for one patient who experienced amenorrhea post-treatment. However, this patient’s FSH and estradiol levels were within the normal range on the third cycle day, especially when the uterine volume exceeded 240 cm^3^ [23].

The use of artificial ascites during PMWA has been evaluated for its ability to improve the feasibility, efficacy, and safety of ablation. Hai et al. [41] retrospectively compared patients who underwent PMWA with and without artificial ascites. They aimed to facilitate the electrode path to the lesion and compare the efficacy with PMWA alone. The authors found that the use of artificial ascites significantly improved the electrode course to the lesion. Moreover, the mean ablation time and the median NPV ratio were almost identical in the two groups. No notable complications occurred. Finally, the symptom pain scores were significantly lower after than before treatment in the group treated with PMWA and artificial ascites [41].

In the above-cited randomized study by Lin et al. [20], the researchers evaluated the effectiveness and safety of treating symptomatic uterine adenomyosis by PMWA versus ultrasound-guided RFA. There was a considerable difference in the ablation time between the PMWA and RFA groups (16.3 and 37.5 min, respectively), with a similar percentage of ablation of uterine adenomyosis. Moreover, no significant differences were found in the percentage of uterine volume regression, dysmenorrhea and menorrhagia relief, or adverse effects between the groups. Both methods led to shrinkage of the lesions and a volume decrease in the entire uterus [20].

A non-percutaneous approach to MWA has also been investigated. Kanaoka and Imoto [51] researched the efficacy of transcervical interstitial microwave irradiation of adenomyotic lesions at Iseikai Hospital in Osaka, Japan. At the 3-month follow-up, the average hemoglobin level had increased by 4.6 g/dL. Furthermore, at the 3- and 12-month follow-ups, the uterine body volume had significantly decreased by 53% and 52%, respectively, compared with pretreatment. The menorrhagia and dysmenorrhea scores improved as well [51].

#### 3.3.2. Multiple Microwave Endometrial Ablation (mMEA)

A recently developed procedure related to PMWA is multiple microwave endometrial ablation (mMEA). This approach is a form of microwave ablation that can be repeated twice or even three times in the same region, focusing on adenomyosis-induced menorrhagia. Although the operating time for mMEA is longer than that for a single MEA procedure, evidence has shown that there is an increased satisfaction rate compared with a single procedure. The disadvantage is that a second ablation increases the risk of complications, especially the likelihood of heat damage in the intestinal area. Additionally, some patients’ conditions are refractory to a single MEA procedure. Therefore, since 2013, such patients have undergone triple ablation in accordance with changes to the 2012 MEA practice guidelines [28]. Although this has resulted in an improvement in the mean hemoglobin level, limited recurrence of bleeding, and a slight improvement in the mean visual analog scale score, no reports before 2015 addressed the safety and efficacy of triple MEA. Nakamura et al. [48] found much more considerable improvement in both the visual analog scale score and patient satisfaction after mMEA. Their study showed no evidence of serious complications as long as the endometrium was >1 cm thick. The authors concluded that mMEA provides advantages over a single treatment for menorrhagia [48].

Further support for the use of MEA was provided by Kakinuma et al. [46]. The researchers reported its minimal invasiveness in managing hypermenorrhea, uterine fibroids, and organic diseases such as uterine adenomyosis. MEA was performed after patient preparation, including cervical and endometrial examinations to exclude malignancies. One patient developed coagulation necrosis due to thermal denaturation of the endometrium and myometrium. There was no recurrence of bleeding 4 years post-treatment. In another patient, the bleeding was immediately stopped after an emergency MEA was performed. Further examination revealed that the bleeding had been caused by endometrial cancer, for which the patient underwent surgery. In a third patient, MEA was also effective after 4 months of recurrent vaginal bleeding caused by atypical endometrial hyperplasia [46].

#### 3.3.3. PMWA: Safety and Drawbacks

The complication rate of PMWA was low in the above-mentioned study by Yang et al. [23]. Additionally, ovarian function continued to be regular after therapy, confirming the procedure’s safety concerning this aspect [23]. Regarding fertility, the FSH and estradiol levels did not considerably differ before and after PMWA. Thus, ovarian function was not adversely affected. A weakness of this study is the lack of investigation of AMH [47]. The addition of artificial ascites to PMWA has been shown to have a high feasibility rate and to help separate the uterus from nearby organs, therefore protecting them from thermal injury [47]. Fortunately, the cooling effect does not reduce the efficacy of PMWA; thus, no additional energy is required to complete the ablation.

## 4. Peri-Procedural Complications

Peri-procedural complications have been discussed separately for each procedure above-presented. A brief summary of these complications has been provided in Table 4.

## 5. Discussion and Conclusions

The current literature supports thermal ablation as a conservative treatment option for adenomyosis. Despite the promising efficacy and safety data for HIFU, PMWA, and RFA, there is currently no clear consensus on the preferred method for thermal ablation of adenomyotic lesions.

After examining these three image-guided thermal ablation techniques for adenomyosis, it can be concluded that all three are effective and safe, with minimal invasiveness and a low risk of complications. Concerning efficacy, their positive outcomes include uterus volume reduction, shrinkage of adenomyosis lesions, and symptom relief. More specifically, HIFU achieves a higher volume reduction, although the process duration is longer than that in the other two methods. Additionally, HIFU, as a noninvasive technique, works at a lower depth; thus, the location and characteristics of the lesions are critical. The benefits of PMWA include a consistently higher tissue temperature, an increased ablation volume, and a shorter operative time; however, its sensitivity varies depending on the tissue type. Finally, because adenomyosis tends to be refractory to therapy, RFA appears to be the most effective technique regarding the recurrence rate.

In terms of safety, thermal ablative techniques do not negatively affect fertility. To date, no considerable effects on ovarian function or fertility rates have been observed after PMWA. Researchers have emphasized its potential usefulness as an alternative therapeutic option for long-term positive effects on dysmenorrhea, menorrhagia, and life-threatening hemorrhage [29].

Another consideration is the mean ablation time, which depends on the dimension of the target lesion but is considerably shorter for PMWA and RFA. In the study by Lin et al. [20], the mean ablation time was 16.3 ± 4.9 min in the PMWA group and 37.5 ± 6.2 min in the RFA group, whereas it was only 2.6 ± 0.6 h in a recent series by Cai et al. [52].

The optimal thermal ablative technique should be chosen based on the lesion characteristics, the patient’s characteristics and desires, and infrastructure capabilities. Furthermore, because these therapies are relatively new, there is widespread agreement among experts that higher-quality studies are needed in the near future. Such studies will provide more consistent data for clinical practice and decision-making.

## Figures and Tables

**Table 2:** Summary of studies on RFA ablation technique for adenomyosis.

Radiofrequency Ablation				
Article Title	First Author, Year	Study Type and Location	Number of Patients	Main Results
Ultrasound-guided transvaginal radiofrequency ablation combined with levonorgestrel-releasing intrauterine system for symptomatic uterine adenomyosis treatment	Hai, 2021 [40]	Retrospective study China	72 patients (January 2013–January 2016)	Ultrasound-guided RFA used with an LNG-IUS was a simple, safe, and effective alternative for the treatment of symptomatic adenomyosis. Specifically, the uterine volume significantly decreased with an average reduction rate of 55%.
Ultrasound-guided transcervical radiofrequency ablation for symptomatic uterine adenomyosis	Hai, 2017 [41]	Retrospective study China	87 patients (January 2013–October 2015)	This is the first study to assess the safety and efficacy of ultrasound-guided RFA for adenomyosis treatment to date and the first to present the various post-treatment changes in the intrauterine cavity.
Laparoscopic radiofrequency thermal ablation for uterine adenomyosis	Scarperi, 2015 [21]	Longitudinal evaluation Italy	23 patients treated with thermal ablation by RFA	Significant reduction in adenomyosis volume on US and in dysmenorrhea VAS score at 3-, 6-, 9-, and 12-month follow-ups.
Midterm outcome of radiofrequency thermal ablation for symptomatic uterine myomas	Ghezzi, 2007 [42]	Evaluation study Italy	25 patients	RFA of symptomatic fibroids appeared to be a valid alternative to major surgery, with durable symptom relief for most patients and a low recurrence rate at midterm.
Effects of different treatment methods on clinical efficacy and fertility outcomes for patients with adenomyosis	Chu, 2024 [43]	Trial study China	140 patients	Ultrasound-guided RFA in combination with leuprorelin acetate is effective in adenomyosis, which can effectively relieve clinical symptoms, protect postoperative ovarian function, reduce reccurence rate, alleviate pain, and improve quality of life.
Radiofrequency ablation for adenomyosis	Dedes, 2023 [39]	Systematic review Switzerland		RFA is a minimally invasive and organ-preserving treatment in patients with symptomatic adenomyosis. It results in clinically meaningful improvement of adenomyosis-related pain in the short term.

RFA: radiofrequency ablation; US: ultrasound scan; VAS: visual analog scale; LNG-IUS: levonorgestrel-releasing intrauterine system.

**Table 3:** Summary of studies on PMWA technique for adenomyosis.

Percutaneous Microwave Ablation				
Article Title	First Author, Year	Study Type and Location	Number of Patients	Main Results
Ultrasound-guided percutaneous microwave ablation of adenomyosis: a narrative review	Zhang, 2021 [22]	Narrative review China	Thirteen studies involving 736 patients treated with MWA ablation, published from January 2000 to June 2021, retrieved from reliable databases	Ultrasound-guided percutaneous microwave ablation therapy was found to be a feasible, safe, and effective technique for the treatment of adenomyosis and showed potential for clinical application and promotion.
Comparison between microwave ablation and radiofrequency ablation for treating symptomatic uterine adenomyosis	Lin, 2020 [20]	Prospective, randomized, parallel controlled clinical trial China	133 patients (October 2015–October 2017)	The safety and effectiveness of PMWA and US-guided RFA in the treatment of uterine adenomyosis were similar. However, the mean ablation time of PMWA was shorter than that of US-guided RFA.
Considerations for performing microwave endometrial ablation (MEA)—three cases with abnormal test results of endometrial tissue discovered by chance when performing MEA	Kakinuma, 2020 [46]	Case studies Japan	3 patients	Before MEA, even if malignant endometrial diseases are excluded before surgery, a detailed examination is needed because of the potential for malignant endometrial diseases. The situation needs careful monitoring, including postsurgically.
Percutaneous microwave ablation with artificial ascites for symptomatic uterine adenomyosis: initial experience	Hai, 2017 [47]	Evaluation study China	25 patients (May 2015–May 2016)	Percutaneous microwave ablation with artificial ascites was feasible, safe, and efficient in improving access to adenomyosis treatment.
Efficacy of multiple microwave endometrial ablation technique for menorrhagia resulting from adenomyosis	Nakamura, 2015 [48]	Comparative evaluation, Japan	18 patients	Multiple MEAs more effectively managed adenomyosis-induced menorrhagia and reached a higher satisfaction rate than single MEAs.
Ultrasound-guided percutaneous microwave ablation for adenomyosis: efficacy of treatment and effect on ovarian function	Yang, 2015 [23]	Evaluation study China	142 patients	US-guided PMWA was an effective treatment method for adenomyosis without significant effects on ovarian function and fertility. It was recommended to replace hysterectomy in reproductive-age women.
Transcervical interstitial microwave ablation therapy for the treatment of adenomyosis: a novel alternative to hysterectomy	Kanaoka, 2014 [49]	Evaluation study Japan	33 patients	Menorrhagia and dysmenorrhea caused by deep adenomyosis were effectively relieved. Uterine body shrinkage was achieved. Low cost, short hospitalization, and ease of surgery were acceptable for both patients and gynecologists. TCMAM with MEA was found to be a promising alternative to hysterectomy for adenomyosis treatment.
Feasibility study on energy prediction of microwave ablation upon uterine adenomyosis and leiomyomas by MRI	Xia, 2014 [46]	Feasibility study China	63 patients: 49 patients with uterine leiomyomas and 14 patients with adenomyosis (June 2011–December 2012)	When the unit volume of lesions was ablated, uterine adenomyosis lesions required more energy than uterine leiomyomas. Hyperintense uterine leiomyomas needed more energy than hypointense lesions.

PMWA: percutaneous microwave ablation; RFA: radiofrequency ablation; US: ultrasound scan; TCMAM: transcervical microwave adenomyolysis; MEA: microwave endometrial ablation; EMJ: endometrial junction; NPL: non-perfused lesion.

**Table 4:** Summary of peri-procedural complications.

HIFU	RFA	PMWA
- Vaginal discharge - Lower abdominal pain - Skin necrosis - Anterior abdominal wall muscle injuries - Internal organ damage (thermal injury of uterus, peritoneum, bowel)	- Vaginal discharge - Lower abdominal pain - Low fever - Endometrial thermal injury - Itrauterine adhesions	- Post-treatment amenorrhea - Temporary increase in CA-125

HIFU: High-intensity focused ultrasound; PMWA: Percutaneous-Microwave-Ablation.
